# An Atypical Presentation of Polyarteritis Nodosa: Case Report and Review of the Literature

**DOI:** 10.7759/cureus.14197

**Published:** 2021-03-30

**Authors:** Sri Harsha Boppana, Nageswara R Dulla, Bryce D Beutler, Sujatha Pitani, Ratinder Kaur

**Affiliations:** 1 Internal Medicine, University of Nevada Reno School of Medicine, Reno, USA; 2 Internal Medicine, Mythri Multispecialty Hospital, Guntur, IND; 3 Rheumatology, Arthritis Consultants, Reno, USA

**Keywords:** cutaneous polyarteritis nodosa, vasculopathy, polyarteritis nodosum, rheumatoid vasculitis, aneurysm

## Abstract

Polyarteritis nodosa (PAN) is a rare multisystem vasculopathy that predominantly affects medium-sized arteries. Involvement of the cardiac and/or pulmonary vasculature may be fatal. We describe a 67-year-old Japanese male who presented with multiple coronary artery aneurysms and subsequently developed lower extremity gangrene. A diagnosis of PAN was established based on the correlation of clinical presentation and laboratory and imaging findings. In addition, we review other manifestations of PAN and differential considerations for this rare but potentially lethal condition.

## Introduction

Polyarteritis nodosa (PAN) is a rare multisystemic vasculopathy with an incidence of approximately 3 to 4.5 cases per 100,000 people annually in the United States [1]. Medium-sized arteries are typically affected. PAN is distinct from other vasculopathies in that it is not associated with antinuclear or antineutrophilic cytoplasmic antibodies (ANCA) [2]. The most commonly involved organ systems include the gastrointestinal tract, kidneys, and joints. Cardiopulmonary involvement is rare but may be fatal.

The clinical presentation of PAN is variable and may include fevers, chills, weight loss, and hypertension. Laboratory studies typically show elevated erythrocyte sedimentation rate (ESR); autoimmune serologies for antinuclear antibody, rheumatoid factor, and ANCA are typically negative [3, 4]. Diagnosis can be established based on clinical features and computed tomography angiography, which may show aneurysmal and non-aneurysmal dilatation of medium-sized vessels [5-7]. 

We report an atypical presentation of PAN characterized by cutaneous and cardiac symptoms. In addition, we review the literature on PAN and discuss diagnosis and management of this uncommon but potentially deadly condition.

## Case presentation

**Figure 1:**
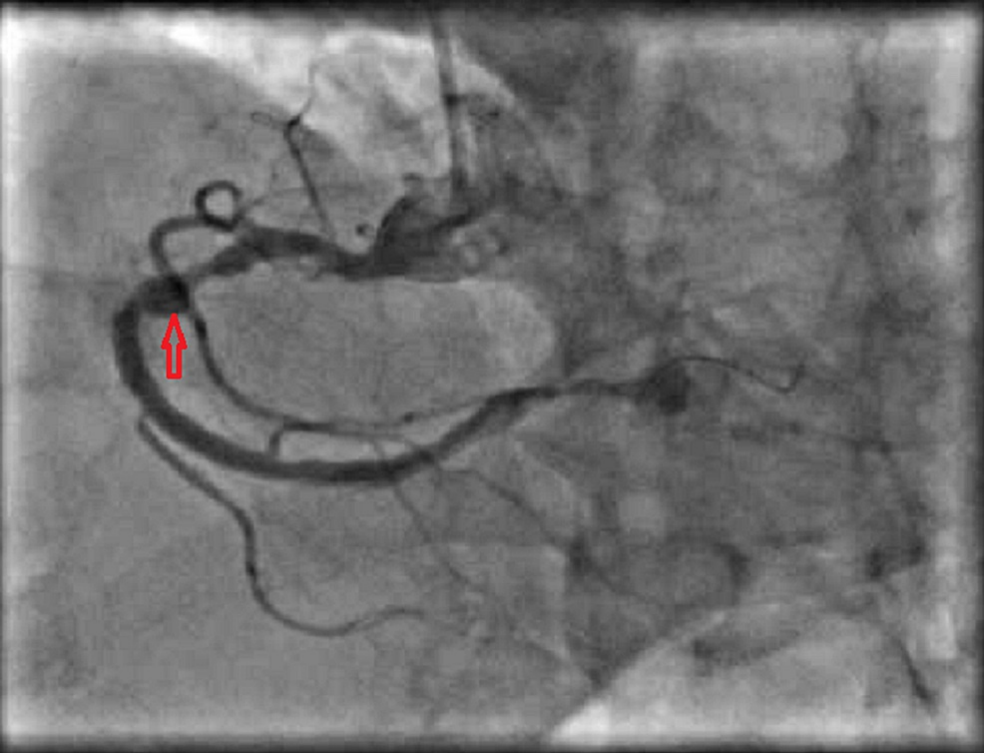
Coronary angiography demonstrating a right coronary artery aneurysm (arrow)

A 67-year-old Japanese male with a past medical history significant for primary biliary cirrhosis, chronic kidney disease stage 3, hypertension, and dyslipidemia presented to hospital with left side chest pain which was insidious in onset, gradually progressive, increasing in intensity and frequency, from past 10 days and radiating to the medial side of the left arm. He was not a known diabetic and was a non-smoker. On presentation, electrocardiography (ECG) was done which showed ST elevation in inferior leads along with sinus bradycardia with heart rate in 30’s. He was taken emergently to the catheterization lab, where it was found that he had complete occlusion of distal right coronary artery (RCA) along with 80% stenosis of proximal RCA, resulting in the placement of three drug-eluting stents to the above (Figure 1).

Incidentally, multiple coronary artery aneurysms were also noted at that time. He was started on aspirin and clopidogrel for coronary artery disease status post stenting and was discharged home once he was medically stable.

**Figure 2:**
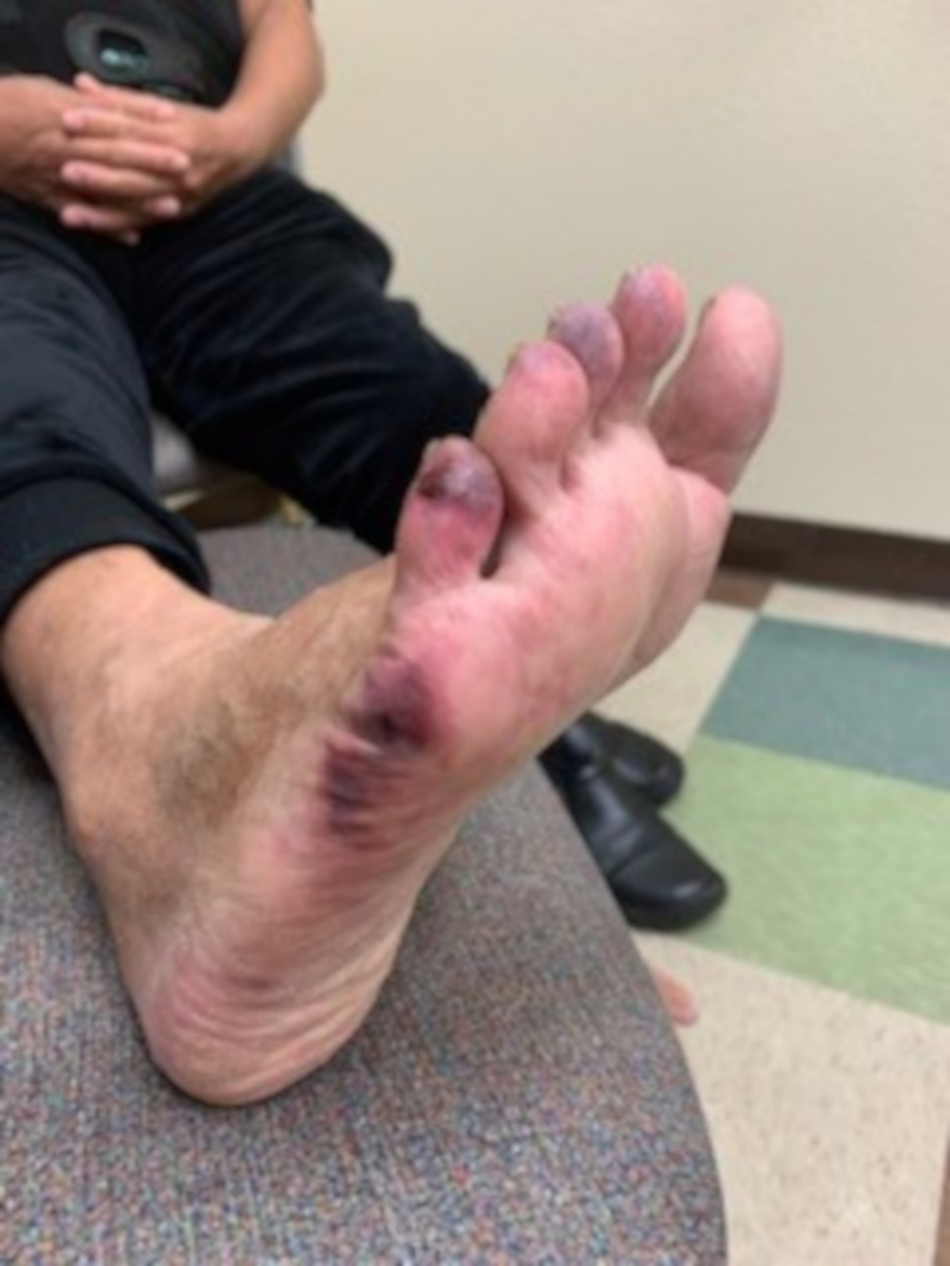
Exquistely tender, dusky-appearing toes of the patient

He presented again to the emergency room, a month later with complaints of substernal chest tightness of 2 hours duration and ECG showed sinus bradycardia with a heart rate of 56 and anterior ST elevation, was taken again emergently to the catheterization lab for left heart catheterization and found to have distal RCA stent thrombosis and underwent successful coronary angioplasty. He informed that he was only taking clopidogrel, hence stent thrombosis was thought to be from non-adherence to dual antiplatelet therapy. After discharge from the hospital, he started noticing progressively worsening severe bilateral lower extremity pain along with gangrenous appearing little toes of both feet and dusky appearance of remaining toes (Figure 2).

**Figure 3:**
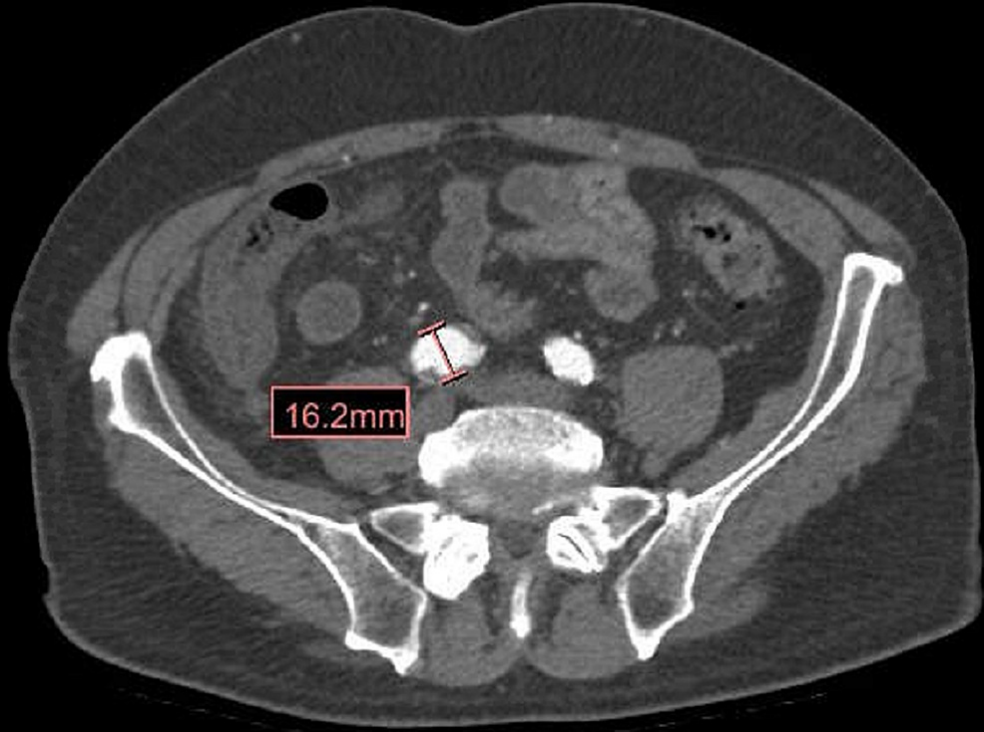
CT angiogram aorta demonstrating right common iliac artery aneurysm measuring 1.6 cm

**Figure 4:**
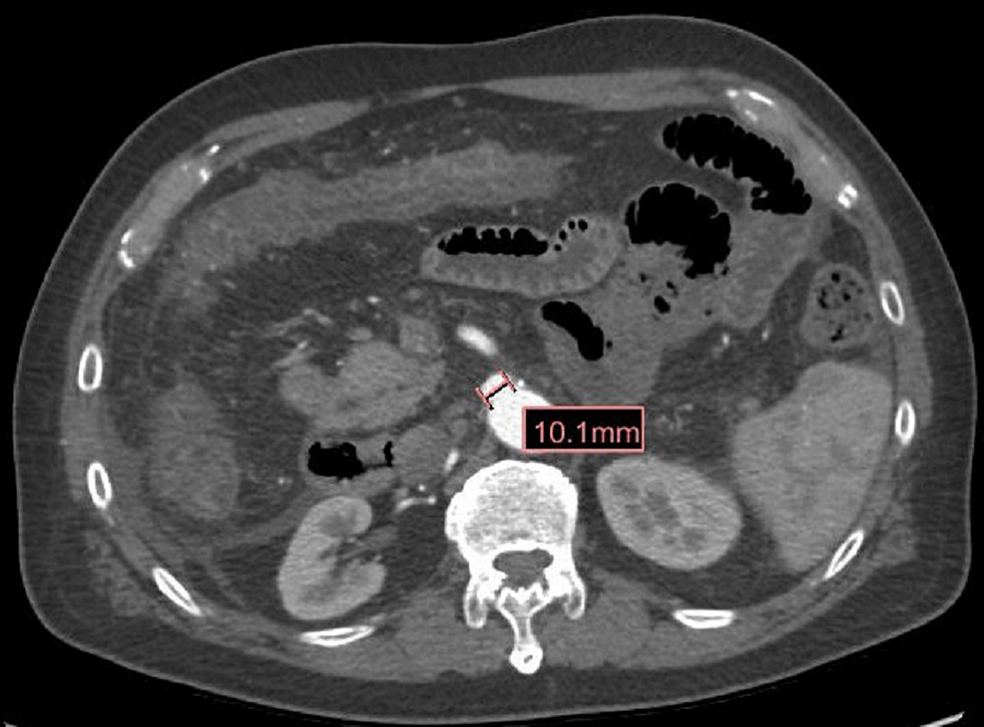
CT angiogram aorta demonstrating saccular aneurysm measuring 1 cm at origin of superior mesentric artery

Lower extremity arterial doppler was negative, which ruled out peripheral vascular disease. So, in suspicion of ongoing vascular disease, CT angiogram of the abdominal aorta was done which showed 1.6 cm right common iliac artery aneurysm along with 1 cm saccular aneurysm at the origin of the superior mesenteric artery but with no significant vessel narrowing or presence of emboli (Figures 3, 4).

Considering the peripheral artery aneurysms and coronary aneurysms, differentials included sequelae of Kawasaki disease along with systemic vasculitis and was referred to rheumatology.

Lab investigations showed negative serologies including ANCA and antinuclear antibody. Markers of inflammation, erythrocyte sedimentation rate was elevated to 31 mm/hr and C-reactive protein was normal along with normal complement levels and hepatitis serologies. After excluding viral causes - including hepatitis B and C and HIV - the patient was started on immunosuppressive therapy with oral prednisone at a dose of 1 mg/kg, which he took for a brief period with improvement in gangrenous symptoms, and was eventually tapered off. He was also planned to be started on maintenance therapy with cyclophosphamide but had issues with approval from his insurance and could not afford to buy the medication. Other alternative options were discussed, but the patient was not inclined as his symptoms improved. Considering his presentation, it is believed he has underlying systemic vasculitis such as polyarteritis nodosa.

## Discussion

Myocardial infarction is not uncommon to find in a patient having dyslipidemia and chronic kidney disease (CKD) stage 3. Peripheral vascular disease is uncommon to find in patients who are nonsmokers and nondiabetic, which commonly presents as bilateral intermittent claudication of lower limbs, with the only positive risk factors for this patient being age and dyslipidemia. PAN is a necrotizing vasculitis involving medium to small vessels of most organs in the body but spares the lungs, glomerular capillaries, and the venous system [6]. Diagnosis of PAN is clenched by considering the whole clinical picture with abnormal angiographic findings of thrombosis and aneurysms at branching points with no specific diagnostic investigations.

Microscopic polyangiitis was ruled out after findings of negative ANCA, absence of evidence of glomerulonephritis, and lung involvement. As the patient was of Japanese descent, Kawasaki was in the differential first but the absence of lymphadenopathy, age of presentation, and response to steroids rule it out. There are multiple case reports of PAN among adults, who presented with symmetrical progressive intermittent claudication of lower limbs [7-15].

Our patient presented with myocardial infarction and digital gangrene of lower limbs with elevated inflammatory markers along with negative ANCA. In one of the above cases, the patient has responded well to aggressive immunosuppression [11].

In the two cases reported by Ninomiya et al. [9] and Heron et al. [10], there were demonstrable characteristic microaneurysms similar to what was seen in the case detailed in this report. But Shukla and Aggarwal [11] and DeGolovine et al. [12] described two cases with segmental stenosis of large vessels without any aneurysmal dilatations.

In an additional study of pediatric PAN by Eleftheriou et al., authors found aneurysms in nearly half of the patients, and the remaining patients demonstrated arterial cutoffs and/or stenoses on angiography. Features of lower limb vascular insufficiency were not identified [4].

## Conclusions

Diagnosis and treatment of PAN are important, which should be considered in a patient with aneurysms and stenosis even in coronary arteries regardless of age of presentation, after other causes have been ruled out.
